# Response of the cytoplasmic and membrane proteome of *Corynebacterium glutamicum *ATCC 13032 to pH changes

**DOI:** 10.1186/1471-2180-8-225

**Published:** 2008-12-17

**Authors:** Mónica Barriuso-Iglesias, Daniela Schluesener, Carlos Barreiro, Ansgar Poetsch, Juan F Martín

**Affiliations:** 1 Instituto de Biotecnología de León (INBIOTEC), Parque Científico de León, Av. Real, 1, 24006. León, Spain; 2 Lehrstuhl für Biochemie der Planzen, Ruhr Universität Bochum. ND 3/130. Universitätsstrasse 150, 44801. Bochum, Germany; 3 Departamento de Biología Molecular de la Universidad de León, Facultad de Ciencias Biológicas y Ambientales, Campus de Vegazana, s/n, 24071. León, Spain

## Abstract

**Background:**

*C. glutamicum *has traditionally been grown in neutral-pH media for amino acid production, but in a previous article we reported that this microorganism is a moderate alkaliphile since it grows optimally at pH 7.0–9.0, as shown in fermentor studies under tightly controlled pH conditions. We determined the best pH values to study differential expression of several genes after acidic or basic pH conditions (pH 6.0 for acidic expression and pH 9.0 for alkaline expression). Thus, it was interesting to perform a detailed analysis of the pH-adaptation response of the proteome of *C. glutamicum *ATCC 13032 to clarify the circuits involved in stress responses in this bacterium. In this paper we used the above indicated pH conditions, based on transcriptional studies, to confirm that pH adaptation results in significant changes in cytoplasmatic and membrane proteins.

**Results:**

The cytoplasmatic and membrane proteome of *Corynebacterium glutamicum *ATCC 13032 at different pH conditions (6.0, 7.0 and 9.0) was analyzed by classical 2D-electrophoresis, and by anion exchange chromatography followed by SDS-PAGE (AIEC/SDS-PAGE). A few cytoplasmatic proteins showed differential expression at the three pH values with the classical 2D-technique including a hypothetical protein *cg*2797, L-2.3-butanediol dehydrogenase (ButA), and catalase (KatA). The AIEC/SDS-PAGE technique revealed several membrane proteins that respond to pH changes, including the succinate dehydrogenase complex (SdhABCD), F_0_F_1_-ATP synthase complex subunits b, α and δ (AtpF, AtpH and AtpA), the nitrate reductase II α subunit (NarG), and a hypothetical secreted/membrane protein *cg*0752. Induction of the F_0_F_1_-ATP synthase complex β subunit (AtpD) at pH 9.0 was evidenced by Western analysis. By contrast, L-2.3-butanediol dehydrogenase (ButA), an ATPase with chaperone activity, the ATP-binding subunit (ClpC) of an ATP-dependent protease complex, a 7 TMHs hypothetical protein *cg*0896, a conserved hypothetical protein *cg*1556, and the dihydrolipoamide acyltransferase SucB, were clearly up-regulated at pH 6.0.

**Conclusion:**

The observed protein changes explain the effect of the extracellular pH on the growth and physiology of *C. glutamicum*. Some of the proteins up-regulated at alkaline pH respond also to other stress factors suggesting that they serve to integrate the cell response to different stressing conditions.

## Background

Since its discovery [1], *Corynebacterium glutamicum *ATCC 13032 is one of the most important organisms used industrially to produce not only various amino acids, but also vitamins, organic acids, proteins and diverse raw chemicals [2-4]. This strain is a facultative anaerobic Gram-positive soil bacterium with a high G+C content. Recently, the entire genome sequence of *C. glutamicum *ATCC 13032 has been determined in parallel in Japan [5] and in Germany [6]. Availability of the genome sequence allowed considerable progress in gene organization studies, transcriptional analyses [7-11], proteome [12,13] and evolutionary studies of this strain [14].

Among the most influential environmental factors in the growth and physiology of bacteria are temperature and extracellular pH. Due to the fluctuations of the pH in the nature microorganisms have developed throughout the evolution diverse adaptation strategies to minimize the damage induced by the stress of an acid or basic environment [15]. Most of the studies in this field were made in *E. coli *[16] or *Bacillus *species [17-19]. For example, in enteric bacteria changes in pH contribute to disease. Low pH enhances expression of numerous virulence factors such as the *ToxR-ToxT *virulence regulon in *Vibrio cholerae *[20], the *phoP-phoQ *regulon of *Salmonella enterica *[21], and the pH 6 antigen of *Yersinia pestis *[22]. *In E. coli *low pH accelerates acid comsuption and proton export, while coinducing oxidative stress, possibly through increased production of oxygen radicals. On the other hand, high pH accelerates proton import while repressing the energy-expensive systems of flagellar biosynthesis and chemotaxis [16]. In Gram-positive soil bacterium *Bacillus subtilis *the σ^W ^regulon is induced as a response towards an alkaline shock [19].

In *C. glutamicum*, proteomic methods have been successfully used in several studies to analyze physiological responses to different stimuli such as ammonium or nitrogen starvation [23,24], growth on glucose or acetate [25], heat shock [10], exposure to herbicides [26], or more recently the metabolic response in a H^+^-ATPase-defective mutant [27]. While numerous responses to different stresses are well studied, the pH-stress response in *C. glutamicum *remains poorly understood, and relatively few proteomic studies have been done in this field, most of them in enteric bacteria [28,29].

The technique most frequently used in *C. glutamicum *for proteome analysis has been the 2D-electrophoresis in combination with mass spectrometry [13,30,31]. In most cases this technique works satisfactorily for cytoplasmatic proteins but not for membrane proteins, due to extremes in pI and in hydrophobicity observed for many membrane proteins. Therefore, intense efforts have been made to develop diverse alternative methods for the analysis of integral membrane proteins [32-34]. One technology particularly useful for the separation and relative quantification of intact membrane proteins is the consecutive separation with anion-exchange chromatography and SDS-PAGE [35,36].

Using a combination of these techniques we observed significant patterns of membrane protein changes in response to pH variations and showed an overlapping of pH stress with other stress responses.

## Methods

### Bacterial strains and growth conditions

*Corynebacterium glutamicum *ATCC 13032 was grown in trypticase soy broth (TSB) at 30°C and at different pH conditions (pH 6.0 as acidic, pH 7.0 as control, and pH 9.0 as alkaline condition), respectively, in three identical BIOSTAT 5-liter fermentors equipped with automatic pH control. The pH values were maintained at ± 0.1 units of the initial pH conditions. The pH was controlled automatically by dropwise addition of 1 M HCl or 1 M KOH. The cultures were grown aerobically in stirred fermentors. The *C. glutamicum *growth kinetics at pH values were as described previously [9].

### Preparation of cytoplasmic protein extracts

Cells of *C. glutamicum *ATCC 13032 from a 50 ml culture at three different pH conditions, were harvested in the mid-exponential growth phase (OD_600_, 3.5–4.0), by centrifugation for 10 min at 4500 × *g*. Preparation of cytoplasmic protein extracts and 2-DE analysis were conducted as described in [10]. Briefly, the cells were harvested by centrifugation, washed, and disrupted in a Fastprep machine (BIO 101). The disrupted cells were centrifuged at 160000 × *g *to remove cell debris and particulate matter. The cell-free supernatant was treated with Benzonase (Merck) and the proteins were concentrated and precipitated by acetone. Finally the protein pellet was dried and resuspended in rehydration buffer. Protein concentrations of the crude extracts were determined by the Bradford method [37]. Precast IPG strips with linear pH gradients of 4.5 to 5.5, and 4.0 to 7.0, were used for the isoelectric focusing (IEF) step. After the SDS-PAGE second dimension, gel spots were quantified relative to each other by densitometry using the ImageMaster™ 2D Platinum Software (GE Healthcare Life Science). Proteins were regarded as regulated if the corresponding ratios referring to the relative volume of the spots changed more than two-fold and if this regulation pattern was found in all biological replicates.

### Preparation of membrane protein extracts. AIEC/SDS-PAGE (2D-IEC)

*C. glutamicum *membrane protein extracts were prepared by following the method described by Schluesener and co-workers [35] from 1 liter of cells grown in TSB at different pH conditions to the mid-exponential growth phase (OD_600_, 3.5–4.0). Briefly, cells were disrupted in a French Press (Thermo Spectronic) with 4 passages at 20,000 psi. Unbroken cells and cell debris were removed by centrifugation twice at 5,000 × g and 4°C. Membranes were enriched by ultracentrifugation at 100,000 × g and 4°C for 30 min. The resulting pellet was resuspended gently with ice-cold PBS buffer and ultracentrifugation was repeated, and membranes were washed with 2.5 M NaBr to remove membrane-associated proteins. The resulting membrane fraction was solubilized in buffer containing 2% (w/v) ASB-14 and applied onto an anion exchange column (Poros20 HQ material, Applied Biosystems). Fractions eluted from the anion exchange column were precipitated with trichloroacetic acid [38], and SDS-PAGE was performed according to Laemmli [39] using gels with concentrations of 12% acrylamide + bisacrylamide and 1% crosslinking. Gels had a separation length of 20 cm. Proteins were stained with colloidal Coomassie and gels were scanned on an image scanner with the LabScan software (Amersham Biosciences). The scanner was calibrated with a grayscale marker (Kodak), and the same settings were applied for all gels. Scanning was carried out at 300 dpi and 8-bit grayscale. Gel bands were quantified relative to each other by densitometry using the software Scion Image (version 4.0.2; Scion Corporation, ). Proteins were regarded as regulated if they passed at least one of these two criteria: (i) the corresponding ratios referring to the relative volumes of the spots changed more than two-fold and if this regulation pattern was found in all biological replicates; (ii) p-value < 0.1, from Student's t-test (paired, two tailed). In many cases, all or most members of an operon showed similar regulation factors; the succinate dehydrogenase complex and the F_0_F_1_-ATPase operon was particularly consistent.

### In-gel tryptic digestion and MALDI-TOF PMF protein identification

Cytoplasmatic protein spots were excised from gels and digested with modified trypsin (Promega) as described in [11]. Membrane protein bands were excised from the Maxi-size gels, destained following the protocol of Hellman *et al *[40], and were also digested with modified trypsin (Promega). The peptide mass fingerprints were determined with Voyager DE-Pro Instrument in reflector mode (Applied Biosystems). Identification was performed using the MASCOT software [41]. Parameters of the search were set as in [28].

### Western-Blotting of AtpD

Inmunological detection of AtpD was performed using 25 μg of cytoplasmic or membrane protein extracts. Proteins were diluted in loading buffer (62 mM Tris-HCl, pH 6.8, 3% SDS, 5% β-mercaptoethanol, 10% glycerol and 0.01% bromophenol blue), and incubated for 5 min at 95°C. Membrane protein samples were not boiled but incubated for 30 min at 60°C to avoid aggregation. After brief coooling, the mixture was centrifuged (5 min, 12,000 × *g*) and the supernatant was loaded onto the gel. Proteins were analyzed by SDS-PAGE (1 hour, at 30 mA per gel) and electrotransferred onto PVDF membranes applying a current of 1 mA/cm^2 ^of gel at maximum for 1 hour. "Prestained Protein Molecular Weight Marker" (MBI Fermentas), ranging in size between 120 kDa and 20 kDa were used as molecular weight markers. Membranes were blocked by incubating in PBST [137 mM NaCl, 1.5 mM KH_2_PO_4_, 7.9 mM Na_2_HPO_4_, 2.7 mM KCl, 0.5% Tween 20 (v/v)] with 3% nonfat milk powder for 1 h at room temperature. Primary antibody was added to this buffer (1:10,000), and the blot was incubated for 2 h or overnight. Membranes were washed 4 times thoroughly in PBST, and then incubated with the anti-rabbit secondary antibody conjugated to horseradish peroxidase (Sigma-Aldrich). Proteins were detected using p-coumaric acid, 3-aminophtalhydrazide (Luminol, Sigma-Aldrich), and 30% H_2_O_2 _as substrates.

## Results

### Cytoplasmatic proteome changes due to pH adaptation

**Figure 1:**
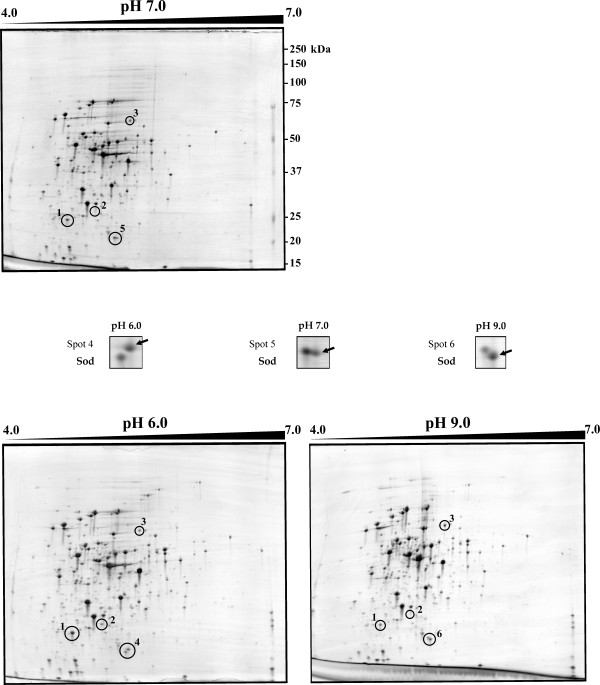
**Comparison of 2D-gels of cytoplasmic proteins of *Corynebacterium glutamicum *ATCC 13032 grown at pH 7.0, control condition (upper panel), versus pH 6.0 and pH 9.0, acid and basic conditions (left and right lower panels, respectively).** The linear pH range used in first dimension was 4.0 to 7.0. In the space between upper and lower panels the spots 4, 5 and 6 identified by MALDI-TOF PMF are shown in more detail. Molecular masses, in kDa, are indicated on the right side of the upper panel.

**Figure 2:**
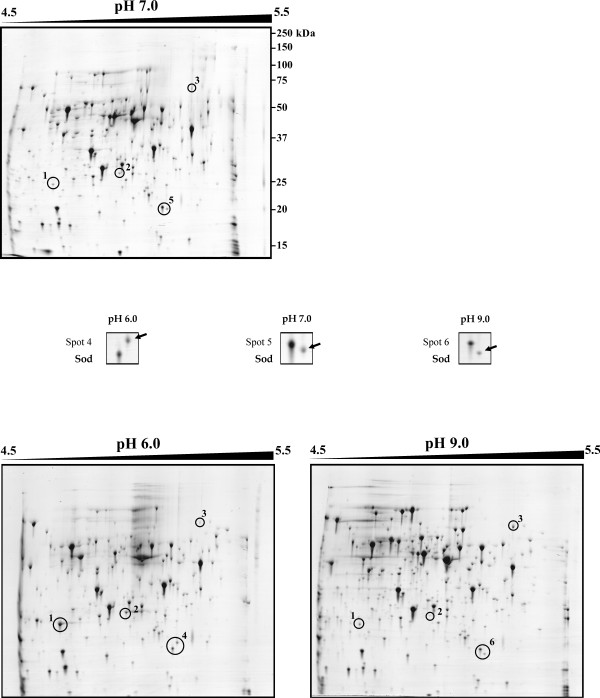
**Comparative expanded (ampholite range 4.5 to 5.5) 2D-gel analysis of *Corynebacterium glutamicum *ATCC 13032 cytoplasmic proteins at pH 7.0, control condition (upper panel), versus pH 6.0 and pH 9.0, acid and basic conditions (left and right lower panels, respectively).** To increase the resolution of the spots, a linear pH range of 4.5 to 5.5 was applied in the first dimension. Protein spots 4, 5 and 6 identified by MALDI-TOF PMF are shown in more detail in the space between upper and lower panels. Molecular masses in kDa are indicated on the right side of the upper panel.

Using three different pH conditions (pH 6.0, pH 9.0 and control pH 7.0) defined in a previous study [9], protein changes in 2D-gels were analyzed from four independent experiments (biological replicates). Initially, precast IPG strips with linear pH gradients of 4.0 to 7.0 [10] were used for the isoelectric focusing step, in which most of the *C. glutamicum *proteins were found within the pI range 4.0–7.0. With this first approach three proteins were detected that showed different abundance in the three pH conditions (Fig. 1). Another protein spot seemed to have different molecular mass at the three pH conditions (indicated as spots 4, 5 and 6 in Fig. 1). To achieve a more detailed resolution from all of these proteins, an ampholite pH range of 4.5 to 5.5 was used (Fig. 2). This 'enlarged 2D-gel' conditions confirmed the same protein spots observed initially. All of these proteins were unequivocally identified by MALDI-TOF PMF analysis (Table 1), and in all cases no more than one protein was found in each gel spot. The two proteins that showed clearly a large increase in response to acidic pH (spots 1 and 2) were identified as: *i) *a hypothetical protein *cg*2797 and, *ii) *ButA which corresponds to L-2.3-butanediol dehydrogenase/acetoin reductase. The only protein (spot 3) that increased clearly in response to alkaline pH was identified as a catalase (KatA). Moreover, modifications by proteolytic processing due to basic pH could be responsible for the occurrence of three protein spots (4, 5 and 6) with identical pI but apparently different molecular mass. These three spots correspond to superoxide dismutase protein (Sod).

**Table 1:** Cytoplasmic proteins whose levels increase or decrease significantly in response to pH shock^*a*^

**Spot**	**Name**	**Accesion no.**	**Function**	**Cal. MW kDa**	**Calc. pI**	**Factor Mowse^*b*^**	**pH 6.0/7.0 ratio**	**S.D.^*c*^**	**pH 9.0/7.0 ratio**	**S.D.^*c*^**	**pH 9.0/6.0 ratio**	**S.D.^*c*^**
**1**	Hypot. protein	*cg*2797	(2R)-phospho-3-sulfolactate synthase (*comA*)	30.3	4.77	96	**↑3.21**	0.06	1.32	0.22	**↓0.42**	0.25
**2**	ButA	*cg*2958	L-2.3- butanediol dehydrogenase/acetoin reductase	27.0	4.51	89	**↑2.18**	0.14	0.85	0.17	**↓0.43**	0.10
**3**	KatA	*cg*0310	Catalase	58.6	5.13	255	**0.73**	0.03	1.45	0.12	**↑2.03**	0.08
**4**	Sod	*cg*3237	Iron/manganese superoxide dismutase	22.0	5.15	83						
**5**	Sod	*cg*3237	Iron/manganese superoxide dismutase	< 22.0	5.15	92						
**6**	Sod	*cg*3237	Iron/manganese superoxide dismutase	< 22.0	5.15	97						

^*a*) ^Only one protein was proposed by MASCOT software for each spot. Arrows indicate up- or down-regulation. In boldface are shown the proteins whose ratios change more than two fold.

^*b*)^Mowse factor, significance level provided by MASCOT software.

^*c*) ^Standard deviation; n = 4

### Membrane proteome changes in response to pH stress

**Figure 3:**
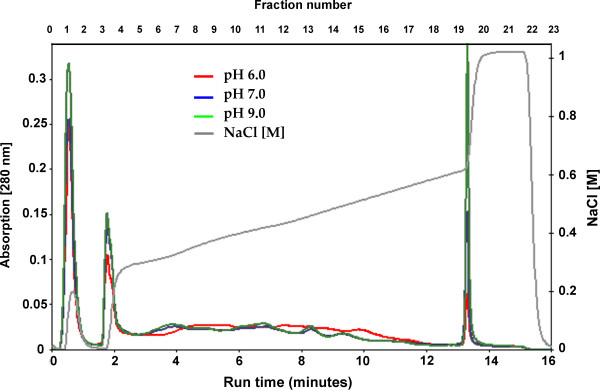
**Elution profile of membrane proteins in Anion-exchange Chromatography (AIEC).** The washed and solubilized *C. glutamicum *membrane fractions at different pH conditions (1.2 mg protein each), were separated using AIEC as first dimension (1.5 ml column packed with PorosHQ20). Proteins were eluted by an increased salt gradient from 0.2 to 0.65 M NaCl in 38 column volumes (cv), followed by a sharp increase up to 1 M NaCl. The flow rate was set to 5 ml/min. Finally the column was washed with 6.5 cv of elution buffer and fractions of 1.5 ml were collected. A_280 _nm is indicated by red, blue and green lines for pH 6.0, pH 7.0 and pH 9.0 samples, respectively. The black line shows conductivity (salt concentration).

**Figure 4:**
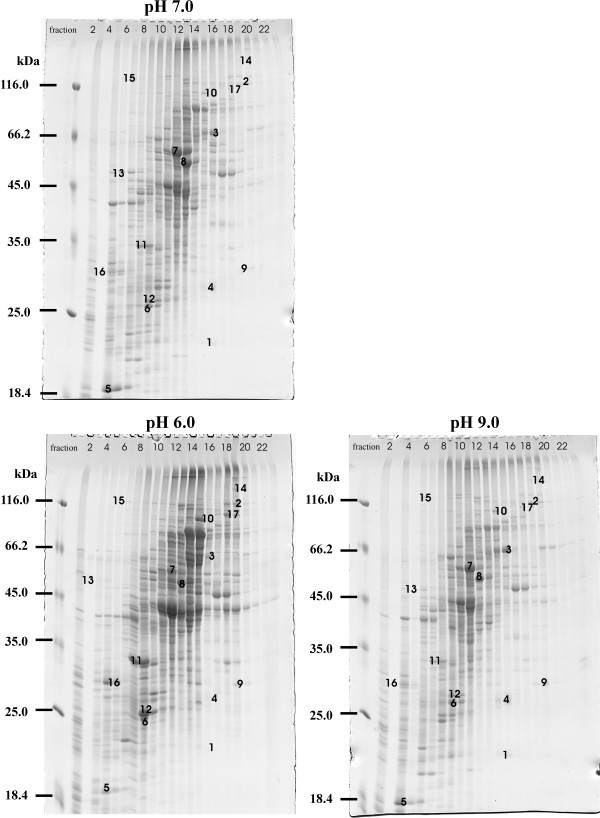
**Separation of the solubilized membrane fractions from *C. glutamicum *ATCC 13032 in control condition (upper panel), versus acidic and basic conditions (left and right lower panels, respectively).** Before separation in the first dimension by AIEC, the membranes were washed twice with 2.5 M NaBr and solubilized in buffer containing 2% (w/v) ASB-14. After the TCA precipitation of the AIEC fractions, SDS-PAGE was used as second dimension in Maxi size gels. Fraction numbers of the AIEC are given at the top of the gels. Numbers indicate the protein spots with differences among the 3 pH values chosen for MALDI-TOF analysis and are listed in Table 1. Molecular masses (in kDa) are shown on the left side of the panels.

A comparative analysis of the membrane proteome was performed with three independent experiments (biological replicates), and using the same acidic and basic pH culture conditions as for cytoplasmatic proteins. Membrane proteins were resolved using a combination of anion exchange chromatography (AIEC) as first dimension, and SDS-PAGE as second one [35]. Fig. 3 shows an AIEC separation of the *C. glutamicum *membrane fractions at different pH values after the washing and solubilization steps. Fig. 4 shows the SDS-PAGE separation in Maxi-size gels of *C. glutamicum *membrane fractions obtained from cultures grown at different pH values. For most of the proteins no differences in expression were visible. At the present time there is no satisfactory software for the comparison of bands in this type of gels, and therefore, the first screening of the gels to choose the protein spots that seemed to increase or decrease their intensity, was performed visually [36]. Several differences were detected by comparing the protein patterns obtained at the different pH values. Seventeen proteins were observed that showed significant changes in response to different pH values (Fig. 4). Six of them (spots 2, 10, 11, 12, 16 and 17) were drastically up-regulated at pH 6.0, while eight proteins (spots 1, 3, 4, 5, 6, 7, 13 and 15) were up-regulated at pH 9.0. Additionally, we observed other three proteins (spots 8, 9 and 14) that change in response to pH variations although the data were not significant enough to define an up-regulation at pH 9.0 (spots 8 and 9), or a regulation at pH 6.0 or 9.0 (spot 14). All of these proteins were unequivocally identified by MALDI-TOF PMF analysis, and in all cases only one protein was found in each spot.

Table 2 summarizes the pH-regulated proteins of the *C. glutamicum *membrane fractions. The six proteins that showed clearly a drastic increase in response to acidic pH (spots 2, 10, 11, 12, 16 and 17) were respectively identified as: *i) *a hypothetical protein (*cg*0896) with 7 transmembrane helices (TMHs), *ii) *the ATP-binding subunit of an ATPase with chaperone activity (ClpC), *iii) *CysK which corresponds with O-acetylserine thiol-lyase, *iv) *L-2.3-butanediol dehydrogenase/acetoin reductase (ButA), *v) *a conserved hypothetical protein *cg*1556, and *vi) *a dihydrolipoamide acyltransferase (SucB).

**Table 2:** Proteins identified in the membrane fractions of *C. glutamicum *ATCC 13032, with their regulation factors, at different pH conditions

								**Regulation factors^*e*^**					
								
**Spot**	**Name**	**Accesion no.**	**Function**	**L^*a*^**	**Calculated MW (kDa)**	**Calculated pI**	**pH induction^*b*^**	**6.0/7.0**	**S.D.^*f*^**	**9.0/7.0**	**S.D.^*f*^**	**9.0/6.0**	**S.D.^*f*^**
**1**	Sdh CD	*cg*0445	Succinate dehydrogenase CD	5	28.2	9.82	pH 9.0	0.68^***g***^	0.15	1.30^***g***^	0.06	**↑2.04**^***g***^	0.52
**2**	Hypothetical protein (7 TMHs)	*cg*0896	---	7	10.9	4.35	pH 6.0	**↑2.02**^***g***^	0.76	1.18	0.16	0.64^***g***^	0.14
**3**	Sdh A	*cg*0446	Succinate dehydrogenase A	a	7.40	5.40	pH 9.0	**↓0.48**^***g***^	0.12	1.26	0.20	**↑2.85**^***g***^	1.07
**4**	Sdh B	*cg*0447	Succinate dehydrogenase B	a	2.65	5.43	pH 9.0	0.61	0.18	1.41^***g***^	0.22	**↑2.52**^***g***^	0.77
**5**	AtpF	*cg*1364	F0F1-type ATP synthase b subunit	a	20.9	4.97	pH 9.0	**↓0.41**^***g***^	0.16	0.86	0.26	**↑2.17**^***g***^	0.24
**6**	AtpH	*cg*1365	F0F1-type ATP synthase delta subunit	a	28.7	5.36	pH 9.0	0.55	0.25	0.85	0.21	↑1.75^***g***^	0.46
**7**	AtpA	*cg*1366	F0F1-type ATP synthase alpha chain	a	58.6	4.77	pH 9.0	0.60^***g***^	0.08	0.88	0.11	↑1.47^***g***^	0.16
**8**	AtpD	*cg*1368	F0F1-type ATP synthase beta chain^***h***^	a	52.4	4.63	---^***c***^	0.61^***g***^	0.10	0.68^***g***^	0.06	↑ 1.12	0.08
**9**	GluB	*cg*2137	Glutamate secreted binding protein	a	31.5	3.89	---^***c***^	0.84	0.36	0.98	0.22	1.30	0.38
**10**	ClpC	*cg*2963	ATPase with chaperone activity (heat shock protein)	a	101.5	4.93	pH 6.0	**↑ 2.91**	2.38	1.00	0.20	0.54	0.24
**11**	CysK	*cg*2833	O-Acetylserine (Thiol)-Lyase	c	32.6	4.68	pH 6.0/pH 9.0	↑1.81^***g***^	0.88	↑1.56^***g***^	0.22	0.91	0.42
**12**	ButA	*cg*2958	L-2.3-butanediol dehydrogenase/acetoin reductase	c	27.0	4.51	pH 6.0	↑1.58^***g***^	0.37	0.78	0.16	0.59	0.25
**13**	Putative secreted/membrane protein	*cg*0752	Uncharacterized BCR	s	49.6	5.06	pH 9.0	**↓0.30**^***g***^	0.03	0.83	0.09	**↑2.79**^***g***^	0.59
**14**	Pks	*cg*3178	Polyketide synthase	a	172.2	4.40	---^***d***^	0.90	0.29	0.63^***g***^	0.11	0.77	0.25
**15**	NarG	*cg*1344	Nitrate reductase 2 alpha subunit	a	139.2	5.74	pH 9.0	0.58	0.35	0.87	0.22	**↑2.63**^***g***^	1.99
**16**	Conserved hypothetical protein	*cg*1556	---	s	33.2	9.70	pH 6.0	↑1.62^***g***^	0.85	1.05	0.14	0.95	0.48
**17**	SucB	*cg*2421	Dihydrolipoamide acyltransferase	c	70.7	4.14	pH 6.0	**↑3.21**^***g***^	1.75	1.38^***g***^	0.09	0.58	0.31

^*a*) ^Localization; c: cytoplasmic, a: membrane-associated, s: secreted, digits indicate numbers of transmembrane helices.

^*b*) ^See discussion.

^*c*) ^The statistical results of three biological culture replicates were not significant enough to define an up-regulation at pH 9.0.

^*d*) ^The statistical results of three biological culture replicates were not significant enough to define a regulation at pH 6.0 or 9.0.

^*e*) ^Regulation factors were obtained from three biological culture replicates for each pH condition. Arrows indicate up- or down-regulation. In boldface are shown the proteins whose ratios change more than two-fold.

^*f*) ^Standard deviation; n = 3.

^*g*) ^Significant difference (t-test, p < 0.1).

^*h*) ^Despite the p-value > 0.1 (0.17), and the 9.0/6.0 ratio is below 2, this protein was regarded as pH regulated by Western-blotting (see Results).

The proteins clearly induced in response to alkaline pH (spots 1, 3, 4, 5, 6, 7, 13 and 15) were respectively identified as: *i) *the three subunits of the succinate dehydrogenase complex (SdhCD, SdhA and SdhB), *ii) *subunits b, δ and α of the F_0_F_1_-ATP synthase complex (AtpF, AtpH and AtpA), *iii) *a hypothetical secreted/membrane protein *cg*0752, and *iv) *the α subunit of the nitrate reductase II (NarG). In this group we have included again CysK, since it is also induced at pH 9.0 but less than under acidic stress (pH 6.0) (see Table 2 and Discussion). The three proteins with poorly significant abundance changes (spots 8, 9 and 14) were identified as: *i) *β subunit of the F_0_F_1_-ATP synthase complex (AtpD), *ii) *a secreted glutamate binding protein (GluB), and *iii) *Pks, which corresponds with a polyketide synthase, a type of enzymes that are frequently involved in secondary metabolism. All of these protein bands were quantified relative to each other using densitometry and the expression factor (up- or down-regulation) found in three independent separation experiments is shown in Table 2.

### Immunological detection of AtpD protein

**Figure 5:**
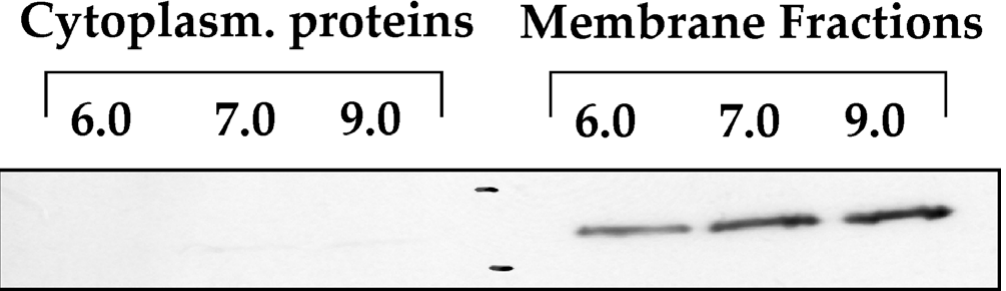
Immunodetection of the AtpD protein by Western blot analysis with antibody anti-AtpD at different pH conditions.

The data on the AtpD protein in the membrane fractions were not significant, unlike the rest of the subunits of the F_0_F_1_-ATP synthase complex identified in this study. To analyze the expression of this protein in more detail western-blot analyses were performed with an antibody against a peptide fragment of Spinach AtpD, using the same samples from the three pH conditions (three biological replicates) described above. The immunological detection of AtpD protein is shown in Fig. 5; a hibridization band of about 53 kDa was found in the membrane fractions of the three cultures, but not in the cytoplasmatic proteins. These bands correspond perfectly with the expected size of the AtpD protein. The intensity of the AtpD band in cells grown at pH 9.0 was 63.2% higher than at pH 6.0 (Fig. 5), and 26.7% higher at pH 9.0 than at pH 7.0. Therefore, we concluded that AtpD is induced at alkaline pH (9.0). These results at the translational level for the F_0_F_1_-ATP synthase beta chain correlate well with those reported at the transcriptional level [9], and with those observed for the other proteins of F_0_F_1_-ATP synthase complex in this work. In summary, all the studied components of the F_0_F_1_-ATP synthase complex are up-regulated at alkaline pH.

## Discussion

Up to date, little information is available concerning the physiology of *C. glutamicum *when grown at different extracellular pH conditions (alkaline or acid), although respiration and membrane transport processes may be affected by these changes in external pH. We used two different strategies to study the analysis of the *C. glutamicum *cytoplasmatic and membrane proteome at different pH conditions.

The first approach was to analyze the proteome by the classical two-dimensional polyacrylamide gel electrophoresis (2-D PAGE). Two proteins were identified as acidic pH induced (Spots 1 and 2, Table 1). A hypothetical protein *cg*2797 was found to be up-regulated 3.2-fold and the L-2.3-butanediol dehydrogenase/acetoin reductase showed a minor up-regulation factor of 2.18 (Table 1). No functional domains were detected in the *cg*2797 protein and homology searches revealed only one protein, ComA [a (2R)-phospho-3-sulfolactate synthase] in the actinobacteria *Rubrobacter xylanophilus *(Rxyl_2563) that show 57% identity. This protein is involved in the Coenzyme M (CoM; 2-mercaptoethanesulfonic acid) biosynthesis, which was thought to be exclusive of methanogenic bacteria until Krum and Ensign [42] also found it in *Xantobacter *Py2 and in *Rhodococcus rhodochrous *B276. In the hyperthermophile marine methanogen *Methanococcus jannaschii*, ComA has been described as the first enzyme required for CoM biosynthesis [43]. This enzyme catalyzes the addition of sulfite to phospho-enolpyruvate forming L-2-phospho-3-sulfolactate, which through several reactions (conducted by the other three enzymes of the pathway, ComB, ComC and ComDE) yields coenzyme M. Since *C. glutamicum *is a Gram-positive bacteria (actinobacteria) related to *Rhodococcus*, it seems to be possible that ComA of *C. glutamicum *is involved in the synthesis of coenzyme M. Moreover, this gene seems to form an operon with another hypothetical protein *cg*2796. There is experimental evidence [44] that this operon is repressed by a transcriptional factor DtxR [45], homologous to the diphtheria toxin repressor DtxR of *C. diphtheriae*, which is involved in regulation of iron metabolism in *C. glutamicum*.

The second pH-regulated protein is the L-2.3-butanediol dehydrogenase/acetoin reductase (ButA). This protein catalyzes the formation of 2,3-butanediol from pyruvate with acetolactate and acetoin as intermediates. Proteomic studies in *C. glutamicum *showed a sharp increase in expression of ButA after heat shock [46], and the same was observed using microarrays (C. Barreiro and J. F. Martín, unpublished results). Interestingly, after heat-shock a significant drop in intracellular pH and an increase of the acetate level was observed in *E. coli *[47]; all three enzymes involved in the 2,3-butanediol synthesis were found to be strongly induced by acetate in several bacteria [48,49]. Besides, in our laboratory another *C. glutamicum *protein identified as Pqo (formerly PoxB) was found moderately up-regulated in response to heat shock [10]. This protein is annotated as pyruvate:quinone oxidoreductase (pyruvate oxydase) that catalyzes the reaction from pyruvate to acetate [50]. These data show that both stressful situations (pH and temperature) up-regulate this route, and the ButA response may serve to integrate different environmental stresses. This is not surprising, since in several bacteria it has been observed that the pH interacts with other environmental factors such as oxygen, temperature or salt concentration.

The catalase (KatA), was found to be up-regulated 2.0-fold at basic pH (pH 9.0/pH6.0 ratio) (Table 1). This protein has also been observed to be up-regulated in transcriptomic studies after heat shock (C. Barreiro and J. F. Martín, unpublished results). In addition, KatA is repressed by the AraC-type regulator RipA under iron limitation conditions [51].

In all these cases, the observed modification of the proteins is the result of an adaptation phenomenon to prolongued incubations at acidic or basic pH. This includes transcriptional up- or down-regulation and post-transcriptional regulation. The latter may include post-translational protein modifications. The superoxide dismutase (Sod) seems to have modifications by proteolytic processing due to basic pH. This situation has also been observed in *S. coelicolor *proteomic studies by Hesketh and co-workers [52], where they reported several other protein spots with lower than expected molecular weights and often different pI values, suggesting modifications by proteolytic processing. Both proteins (KatA and Sod) have been found in most organisms, aerobic and anaerobic, and play a key role in cellular protection against the highly toxic reactive oxygen species (ROS) such as superoxide radical anions (O^2-^), hydrogen peroxide (H_2_O_2_) and hydroxyl radicals (OH^-^). In *C. glutamicum *Sod belongs to the Mn-Sod type, which uses manganese as cofactor. This protein catalyzes the conversion of superoxide radicals, generated indirectly in aerobic respiration, to molecular oxygen and hydrogen peroxide; the latter is broken to water by catalase or peroxidase. In most microorganisms, the Sod protein has a constitutive expression, although in *E. coli *it was observed that the Mn-Sod is an inducible enzyme after exposure to oxygen or to superoxide radicals [53]. Recently, both catalase and superoxide dismutase genes of *Shewanella oneidensis *have been shown to be induced after oxidative stress generated by the extracellular alkaline pH [54]. The data obtained in this study suggest that *C. glutamicum *at an alkaline extracellular pH also develops this protective response against oxidative stress.

The number of proteins identified by the 2D procedure, that show significant changes in expression level as a function of pH, was very low. This observation is not surprising since work in *Bacillus *indicates that only a small number of cytoplasmic proteins change in response to pH stress [55]. This phenomenom reflects the fact that the intracellular pH (cytoplasmatic) does not show drastic changes in response to variations in the extracellular pH [56].

On the other hand, the proteins in the cell surface or those that are partially or entirely exposed to the external environment are likely to be more susceptible to changes in the extracellular pH than the cytoplasmic ones. For this reason the *C. glutamicum *proteome was analyzed by a second approach, the newly reported two-dimensional separation AIEC/SDS-PAGE [35,36,57]. This technique is much more effective than the previous one for resolution of integral membrane proteins (IMPs) and membrane-associated proteins (MAPs). Our results indicate that the proteome obtained by this method at pH 6.0 is very different from that observed at pH 7.0 and 9.0. This difference can be even observed in the elution profiles after the ion exchange chromatography (first step of the AIEC/SDS-PAGE) (Fig. 3). Using this procedure seventeen proteins could be identified, of which two are integral transmembrane proteins (5–7 TMHs), while ten are membrane-associated, two are secreted and three are cytoplasmic (Fig. 4, Table 2).

Two hypothetical proteins (*cg*0896, *cg*1556) were up-regulated 2.0 and 1.6-fold respectively at acidic pH, and other hypothetical secreted/membrane protein *cg*0752 was found up-regulated at alkaline pH about 2.8-fold (Table 2). The first of these three proteins (*cg*0896) is a membrane integral protein, which contains 7 transmembrane spanning domains according to the TMpred program [58]. Very recently, this protein was also found to be induced in *C. glutamicum *during growth on citrate [59], an anion that acidifies the culture medium. This protein may play an important role in acid stress responses.

Protein *cg*1556 that shows homology to members of the DoxD-family was found to increase 1.6-fold at acidic pH and a similar regulation factor (1.7) has been obtained for this protein after heat shock in *C. glutamicum *[46]. DoxD is a subunit of the terminal quinol oxidase present in the plasma membrane of *Acidianus ambivalens *[60]. Under normal growth conditions, the cytochrome *bc*_1_-*aa*_3 _supercomplex is used as terminal electron acceptor (terminal oxidase) and cannot be easily substituted by alternative menaquinol oxidation pathways [61]. Interestingly, Kusumoto *et al*. [62] suggested that a third terminal oxidase exists, besides cytochrome aa3 and cytochrome *bd *[see also [63]]. This DoxD-like protein may be this alternative oxidase.

The protein spot 10 was found to be induced 2.9-fold at pH 6.0 and was identified as a ATP-dependent protease (ClpC). This heat shock protein is one of the components of the Clp holoenzyme (ClpC, ClpP1 and ClpP2). The activation of ClpC might play a role in the degradation of misfolded proteins due to acidification; it may work also as a chaperone to assist the refolding of proteins. The *clpC *and *clpP1P2 *genes of *C. glutamicum *respond also to heat stress, they are positively regulated by the transcriptional factor sigmaH (σ^H^) under a severe heat shock [64]. Moreover, in *C. glutamicum *subsp. *flavum *the transcriptional factor σ^H ^controls the expression of σ^B ^under acidic stress, cold and heat shock [65]. These results suggest a double positive regulation of the expression of *clpC *mediated by the same sigma factor under two stress situations (pH and temperature) that appear intertwined in different microorganisms. The *Bacillus subtilis *heat-shock proteases are induced by heat stress but also by general stress conditions such as exposure to salt or ethanol, or starvation for glucose, phosphate or oxygen [66].

CysK, an O-acetylserine (thiol)-lyase was up-regulated 1.8-fold at basic pH but also showed a modest increase at pH 6.0 with a regulation factor of 1.56. In *C. glutamicum *proteome studies this protein has been identified as a phosphoprotein involved in the biosynthetic pathway of cysteine; it catalyzes the step from O-acetylserine to cysteine. Rey *et al*. [67] identified a repressor protein named McbR which is found to regulate several genes involved in the biosynthesis of methionine and cysteine, such as *cysK*. *E. coli *proteome studies [68] have shown that basic pH induces several amino acid metabolic enzymes, particularly CysK. The up-regulation of the *cysK *gene after an oxidative stress generated by alkaline extracellular pH was also observed in *Shewanella oneidensis *transcriptomic studies [54]. These authors suggest that high pH appears to enhance the cellular demand for cysteine, which may be neeeded to repair oxidative damages. This role of CysK agrees with the results obtained in this work with KatA and Sod that are up-regulted for protection against oxidative stress.

The L-2.3-butanediol dehydrogenase/acetoin reductase protein (ButA) was also found 1.6-fold induced at acidic pH in the membrane protein gels, supporting the results previously obtained in the cytoplasmic gels.

A dihydrolipoamide acyltransferase protein (SucB) was found 3.2-fold up-regulated at pH 6.0. Little is known about the encoding gene *sucB*; Kalinowski *et al*. [6] annotated it by similarity to the 2-oxoglutarate dehydrogenase complex subunit E2o of the citric acid cycle from other organisms, including *odhB *in Gram-positives or *sucB *in *E. coli*.

All three subunits of the succinate-DH complex were up-regulated at alkaline pH with a regulation factor ranging from 2.0 to 2.8 (Table 2). Succinate dehydrogenase is a tightly membrane-bound enzyme catalyzing the oxidation of succinate to fumarate. This enzyme is part both of the TCA cycle and of the aerobic respiratory chain. This enzyme complex consists of three subunits SdhA, SdhB and SdhC; the first two form the membrane-associated cytoplasmatic domains and the third constitutes the integral membrane domain. These three genes are likely to form an operon with two ORF encoding hypothetical proteins (*cg*0448 and *cg*4001) since they are oriented in the same direction and are coordinately up-regulated when *C. glutamicum *is grown on acetate [25] or in citrate [59]. Futhermore, this operon is regulated negatively by the RipA regulator [50] and positively by DtxR transcriptional regulator [44].

Interestingly, the subunits b, δ, α and β of the F_0_F_1_-ATP synthase complex (AtpF, AtpH, AtpA and AtpD) were also found up-regulated at alkaline pH. The induction of AtpD was confirmed by western blot studies. This is in accordance with a previous work in which we established that the abundance of the mRNA of the F_0_F_1_-ATP synthase operon is increased at alkaline extracellular pH [9]. The F_0_F_1_-ATP synthase operon of *E. coli *has been shown also to be induced by alkaline extracellular pH [16]. Furthermore, another recent study showed that this complex is also highly induced (at the mRNA and protein levels) when *C. glutamicum *is grown on citrate [59]. Our hypothesis is that the cells increased the expression of the ATP-ase (a reversible enzyme system) to compensate the alteration of the proton gradient produced by the alkaline pH.

Finally, the alpha subunit of nitrate reductase II named NarG was up-regulated 2.6-fold at alkaline pH. This enzyme is responsible for the nitrate reduction to nitrite and consists of two membrane-associated cytoplasmic domains NarG and NarH bound to a heme-containing membrane anchor (NarI). The nitrate reductase responsible for this reduction is encoded by the *narKGHJI *gene cluster. The reduction of nitrate in *C. glutamicum *by the NarGHI complex under anaerobic conditions generates a electrochemical proton gradient, as happens in *E. coli*, because the quinol oxidation occurs at the outside of the cytoplasmatic membrane and the reduction of nitrate takes place in the cytoplasm [63]. Although *C. glutamicum *has been always regarded as an aerobic microorganism, a work has recently published showing that *C. glutamicum *grows in the presence of nitrate as a terminal electron acceptor [69].

Interestingly, we observed in our data that succinate dehydrogenase and proteins of the respiratory chain, i.e. the alpha subunit of nitrate reductase II and subunits of the F_0_F_1_-ATP synthase complex, showed between 1.4 and 2.8-fold higher abundance at alkaline pH than at acidic pH. The transcriptional regulation of the corresponding ATP-synthase genes [9] correlates well with this increase. In preliminary experiments, we found a membrane-bound NADH dehydrogenase also induced at alkaline pH (data not shown). Some of the observed changes may be indirectly caused by the change in pH and are not directly involved in the maintenance of pH homeostasis as occurs in other bacteria, e.g. it has been reported that in *B. subtilis *alkaline stress results in phosphate starvation [70,71].

## Conclusion

Taking all these results together and the fact that the succinate dehydrogenase is the only enzyme that is part of both the citric acid cycle and the respiratory chain, a possible mechanism of coordinate regulation by alkaline pH of the respiratory chain and the tricarboxylic acid cycle is likely to exist.

In summary, the findings reported in this article suggest a complex interaction between pH, temperature and oxidative stresses. All these stressing factors trigger expression of sets of identical or similar genes that appear to serve to integrate diverse stress signals that in turn activate the cell defences against the stressing conditions.

## Authors' contributions

MBI analyzed the citoplasmic proteome, performed the western-Blotting of AtpD and wrote the manuscript draft. DS analyzed the membrane proteome and was supervised by AP. CB participated in the design of the 2-D PAGE experiments and helped to draft the manuscript. JFM prepared the research project, supervised the experiment work and was responsible for the preparation of the final version of the manuscript.
